# A qualitative examination of the mental health impact of Covid-19 in marginalized communities in Guatemala: The Covid Care Calls survey

**DOI:** 10.1177/00207640211028612

**Published:** 2022-11

**Authors:** Dana Alonzo, Marciana Popescu

**Affiliations:** 1 Graduate School of Social Service, Fordham University, New York, NY, USA; 2 Suicide Prevention Research Program, New York, NY, USA

**Keywords:** Covid-19, mental health, psychological distress, marginalized communities, Guatemala

## Abstract

**Background:**

The mental health impact of Covid-19 from the perspective of individuals experiencing psychological distress during lockdown period in marginalized, high-risk communities remains underinvestigated.

**Aims:**

This study aims to identify key factors related to psychological distress resulting from the Covid-19 pandemic across highly vulnerable districts in Guatemala.

**Methods:**

The Covid Care Calls (CCC) survey was administered to households in 11 districts in Guatemala to gather information about medical, mental health, and psychosocial status during the lockdown period; provide referral for care; and disseminate information on evidence-based protective measures to stem the spread of the virus. The 330 individuals participated the survey. Conventional content analysis was used to analyze survey data.

**Results:**

Most commonly reported mental health issues since the start of the pandemic were anxiety (46%), stress (36%), and exacerbation of pre-Covid-19 mental health conditions (19%). Depression and burnout were equally reported by 12% of participants. Only 2% reported issues with safety in the home. Concerns about catching the virus and economic worries were the most commonly reported sources of psychological distress.

**Conclusion:**

Results of this study indicate a high prevalence of anxiety, stress, and increased prior mental health symptoms resulting from the onset of the Covid-19 pandemic in low-income, high-risk communities across Guatemala. Efforts focused on enhancing coping strategies as well as psychoeducation to address stigma and increase help-seeking for depression are particularly important.

## Introduction

Research over the last several months of the Covid-19 pandemic has established significant negative mental health consequences related to the onset of the virus and subsequent quarantines and lockdown restrictions across the globe. Clinically significant symptoms of anxiety, stress, depression, tension, anger, and fatigue have been identified in multiple countries across Europe, the United States, and Asia (Ordriozola-González et al., 2020; Roma et al., 2020; Terry et al., 2020; Tusev et al., 2020; Wang, Pan, et al., 2020; Wang, Zhang, et al., 2020). However, few studies have focused on Latin America. Only one study could be identified that has examined the impact of the pandemic on mental health in Guatemala. Results indicated that among adults living in highly vulnerable communities characterized by high rates of extreme poverty, crime, familial and gang violence, moderate to high rates of anxiety and stress, and significant increases in pre-existing mental health symptoms were reported.

While this growing body of research has identified the prevalence of mental health issues resulting from the pandemic and related sociodemographic characteristics associated with increased risk of experiencing mental health symptoms, far less studied are the unique factors contributing to the experience of psychological distress during lockdown periods. Even less understood is the impact of the lockdown period on already vulnerable communities in which socioeconomic status, community, and familial circumstances serve as pre-existing stressors, such as those inm Guatemala.

According to the Ministry of Health, as of November, 2020, there have been 111,262 confirmed cases of Covid-19 in Guatemala with 3,821 deaths (Latin American News Dispatch, 2020) Guatemala has experienced a rate of Covid-19 much higher than other Central American countries (Ministry of Health, 2020). Beginning on March 5th, Guatemala entered into a ‘State of Calamity’ resulting in strict lockdown and curfew measures and travel restrictions to cur the spread of the virus. During curfew hours, stores, including grocery stores, were closed and individuals were required to stay at home and those in breach of the curfew were subject to a fine or imprisonment. Except for the transportation of food, water, medicine, gas, and permitted essential services, all other transport was prohibited. Shopping malls were unable to open and food stores will only be permitted to open between the hours of 08:00 and 11:00 from Friday through Sunday. Use of public recreational areas was banned. Additionally, individuals were required to travel by foot and the use of private vehicles was prohibited.

Outside of curfew hours, all individuals were mandated to comply with physical distancing rules, requiring people to stay at least 5 ft apart and utilize facemasks in all public spaces. Violation of the physical distance or facemask regulations would be met with heavy fines. Public events, recreational activities, and large gatherings were canceled/prohibited. International and domestic travel was restricted, if not suspended, and public transport was limited to operate at a 50% capacity (Garda World, 2020).

For many communities around Guatemala City, pre-existing pandemic conditions were already perilous. In these ‘Red Zone’ districts, high rates of extreme poverty, violent and gang-related crime, and substance use, and limited employment and educational opportunities prevail as do high rates of teen pregnancy, delinquency, violence against women, and domestic violence ( Branas et al., 2013; Godoy-Paiz, 2005; Puac-Polanco et al., 2015). Lockdown conditions further limited employment and education as stay at home orders prevented work and regular school attendance. Increased time at home without opportunities for recreational outings and socialization outside of the home increased exposure to and prevalence of family violence with limited potential for intervention (Usher et al., 2020). It is likely that these factors may lead to increased anxiety, stress, and depression (Droit-Volet, et al., 2020; Qiu et al., 2020). Understanding if and how these factors actually do contribute to the experience of psychological distress during lockdown periods will be essential to inform the development of interventions to address the unique mental health issues resulting from the pandemic.

We were unable to identify any study that has specifically explored the factors related to increased psychological distress during the pandemic from the perspective of the individuals struggling with mental health symptoms in highly vulnerable conditions. This study aims to fill that gap and identify the psychosocial factors (i.e. financial hardships, care-giver burden, etc.) contributing to psychological distress (anxiety, stress, burnout, depression, exacerbation of pre-existing mental health conditions, and/or sense of safety) during the lockdown period among individuals residing in highly vulnerable communities across Guatemala.

## Materials and methods

### Sample

In collaboration with Hunger Relief International (HRI) and International Social Work Solutions (ISWS) and with Institutional Review Board approval, we designed and administered a program, Covid Care Calls (CCC), to survey a representative sample of individuals residing in high-risk communities in and around Guatemala City to assess the prevalence of health and mental health issues during the lockdown period; make referrals for medical and mental health care; and curb the spread of Covid-19 by providing psychoeducation on evidence-based protective measures such as physical distancing, regular hand-washing, and mask-wearing. Households/phone numbers were identified by HRI based on their access to local residency data. Oral consent was obtained from participants by interviewers prior to administering the survey.

Individuals from 11 districts participated in Covid Care Calls (CCC) baseline and follow up assessments between June 6th, 2020 and September 30th, 2020. The 11 districts included: El progreso, Guastatoya; Masagua, El Astillero; Mixco; San José Pinula; San Miguel Petapa; Villa Bueva, Villas del Frutal II, zona 6; Villa Nueva; Villa Canales; Guatemala Zona 6; Guatemala, Limón; Guatemala, zona 18. Of the 347 individuals called to complete the baseline survey, 330 agreed to participate and serve as the sample for this study. Follow-up call data was not included in this study. This high participation rate is likely due to the positive reputation of HRI and their community partners in these districts.

### Measures

The CCC survey collected information on the socio-demographic characteristics of participants, health, and mental health status, burnout, their perceived sense of safety within their household; and other emotional/psychological consequences participants identified. Detailed notes were taken by interviewers for each call on information the information shared by participants. Both survey data and the interviewers’ notes were analyzed for this study.

### Sociodemographic characteristics

Participants provided information regarding their sex, age, district of residence, number of children, number of dependent relatives, and household composition.

### Clinical characteristics

All symptoms of psychological distress were assessed using one main single item question with two subsequent follow-up questions asked of those who responded in the affirmative. Single item measures of depression and other psychiatric symptoms have demonstrated both adequate sensitivity and specificity to detect emotional distress in previous studies (Rodríguez-Rey et al., 2019, 2020; Skoogh et al., 2010).

### Anxiety

Participants were asked to respond yes or no to the question, ‘Have you been feeling anxious since the pandemic began?’ If they answered yes, they were than asked two follow-up questions including, ‘On a scale of 1 to 10, 1 being the lowest and 10 being the highest, how anxious do you feel?’. A follow-up question was asked to collect more detail on the factors contributing to participants’ anxiety: ‘Can you share with me the reasons for your anxiety?’.

### Stress

Participants were asked to respond yes or no to the question, ‘Have you been feeling stressed since the pandemic began?’ If they answered yes, they were than asked two follow-up questions including, ‘On a scale of 1 to 10, 1 being the lowest and 10 being the highest, how stressed do you feel?’. To collect more information on the nature and intensity of the stress, participants were asked a follow-up question: ‘Can you share with me the reasons for your stress?’.

### Depression

Participants were asked to respond yes or no to the question, ‘Have you been feeling depressed since the pandemic began?’ If they answered yes, they were than asked two follow-up questions including, ‘On a scale of 1 to 10, 1 being the lowest and 10 being the highest, how depressed do you feel?’ and an open ended question to gather details on the context and factors contributing to the participant’s depression: ‘Can you share with me the reasons for your depression?’.

### Exacerbation of pre-existing mental health symptoms

Participants were asked to respond yes or no to the question, ‘Were you experiencing any mental health symptoms before the pandemic began?’ If they answered yes, they were than asked three follow-up questions including, ‘Have these symptoms been worse since the pandemic?’; ‘On a scale of 1 to 10, 1 being the lowest and 10 being the highest, how much have your symptoms increased?’; and an open ended question to gather details regarding the changes/increases in any of the participants mental heath symptoms: ‘Can you share with me the reasons for your increase in symptoms?’.

### Burnout

Participants were asked to respond yes or no to the question, ‘Have you been feeling burned-out since the pandemic began?’ If they answered yes, they were than asked two follow-up questions including, ‘On a scale of 1 to 10, 1 being the lowest and 10 being the highest, how burned-out do you feel?’. They were then asked a follow-up question to gather details on the context and factors contributing to their burn out: ‘Can you share with me the reasons for your burnout?’.

### Sense of safety

Participants were asked about their sense of safety at home. They were asked to respond yes or no to the question, ‘Do you feel that it is dangerous for you or your loved ones to remain at home?’ If the participant responded yes, a follow-up question was asked, ‘Have there been any physical, verbal or other assaults in the home during the lockdown period?’ The caller would then list other household members and ask if that person was the perpetrator to avoid the participant having to say the name and potentially raise the suspicion of someone listening nearby. Legal and safety resources were then provided to the participant and they were asked if they would like to be called in 2 days for a safety follow-up. Interviewers’ notes/observations represented an added source of information for contextualizing the answers to these questions.

Lastly, participants were asked to share any other feelings they are experiencing that they might not have been asked about or had a chance to share. More specifically, participants were asked, ‘Is there anything else you are experiencing or feeling that you would like to discuss?’ For those who responded yes, they were then invited to share what they were experiencing.

### Data analysis

Participant responses to the mental health items (anxiety, stress, depression, burnout, exacerbation of pre-existing mental health symptoms, and sense of safety) were rated on a scale of 0 (none) to 10 (high). For the purposes of the analyses, all responses to the mental health items rated on the 10-point scale were categorized into three categories including, none (0), low (1–4), or moderate to high (5–10).

Conventional content analysis (Hsye & Shannon, 2005; Morgan, 1993) was used to explore qualitative data obtained from interviewer notes. This is a widely accepted qualitative research method that requires the interpretation of the content of text data through a systematic classification process that involves the coding and identifying of themes. The conventional content analysis approach does not start with preconceived hypotheses or theory-based notions about what kinds of codes or themes will be found, the data drives the codes/themes.

Initial coding was conducted independently by the study PIs. After this first independent coding round, a consensus meeting was held during which like items were pooled (e.g. ‘concerns about getting infected’ and ‘concerns about the virus’) and redundant codes were collapsed (e.g. ‘virus’, and ‘COVID’ collapsed to ‘pandemic’) to create the final categories or domains. In the case of disagreement, category definitions were clarified until agreement was reached until inter-rater consistency for these items reached standards in the literature (i.e. no less than 80%).

## Results

Table 1 reports the sociodemographic characteristics of the sample. Participants were largely female (64%), with an average age of 37 (+11.61). Households ranged in size from 1 person to 15 people, with the majority of participants living with family (96%), having children under 18 years old (51%) and 19% with family over 60 years old living in the home. Eighty-four (25%) participants reported having at least one symptom of Covid-19 and were referred to Government sponsored Covid hotline for assistance.

**Table 1.** Sociodemographic characteristics of the sample.

Sociodemographic characteristic	*N* (%)	Mean (±*SD*)
Age (in years)		37 (±11.61)
Sex		
Female	212 (64)	
Lives with family		
Yes	317 (96)	
Children under 18 years old in the house		
Yes	168 (51)	
Relatives over 60 in the home		
Yes	63 (19)	
Number of household members		
1–3	174 (53)	
4–6	121 (37)	
7–10	28 (8)	
11–15	7 (2)	

The most commonly reported mental health symptom was anxiety. Forty-six percent (46%) of respondents endorsed experiencing increased anxiety since the start of the pandemic, with 51% of those participants rating their anxiety as moderate to high (see Table 2). The qualitative analysis revealed two main categories of anxiety including, (1) illness related concerns, endorsed by 78% of participants with anxiety; and (2) economic concerns, endorsed by 35% of participants with anxiety. In terms of illness related concerns, participants expressed fear of personally getting infected with COVID, concerns around their family member’s becoming ill, and difficulty dealing with the degree of anxiety of their loved one’s around catching the virus. In terms of economic concerns, participants primarily expressed anxiety around their inability to work and having a lack of financial resources to support themselves during the pandemic (see Table 3).

**Table 2.** Symptoms and severity of psychological distress during the pandemic.

Symptom and severity	Participants endorsing symptom *n* (%)
Anxiety	153 (46)
Moderate to high	78 (51)
Low	75 (49)
Stress	118 (36)
Moderate to high	77 (65)
Low	41 (35)
Increase in pre-existing symptoms	64 (19)
Moderate to high	31 (48)
Low	33 (52)
Depression	38 (12)
Moderate to high	7 (2)
Low	33 (98)
Burnout/fatigue	38 (12)
Moderate to high	7 (2)
Low	33 (98)

**Table 3.** Triggers of psychological distress during the pandemic.

Symptom triggers	Participants endorsing trigger *n* (%)
Anxiety	
Illness related concerns	119 (78)
Economic concerns	53 (35)
Stress	
Distress about the future	36 (30)
General frustration	33 (28)
Boredom	31 (26)
Increased work/homework	18 (15)
Increased pre-existing symptoms	
Relational problems	40 (63)
Intrapersonal factors	15 (23)
Depression	
Sadness	7 (41)
Loss of routine	5 (29)
Insomnia	5 (29)
Burnout	
Lack of motivation	17 (100)

Participants shared commented such as:

‘I do not know what is going on with me, but I have noticed that *I am very afraid of getting infected*. I think it all started because I was watching the news to find out what the President was sharing and the number of cases were going up every day. The weeks passed and *I started to feel scared every time I went out in public*. I didn’t want to touch things and if I did I always tried to disinfect my hands with gel. I tried to keep more distance between myself and others on the street and others did the same with me. *Every time I worried more*. I try to keep my house very clean and disinfect it, but I still don’t feel safe because I think I will get infected in my house, too. I’ve been watching the news, but I think seeing all of that is making me sick and creating more fear in me. I am concerned at all time about the contracting the virus - both for myself and my family, and that is why I clean’. (Male, age 50),

‘During this time of the lockdown period spending so much time in the house together, I notice *my parents worried about food*. I asked for help from some family members but *everyone is hurting financially*. There were some people who helped my parents by giving them some food at the beginning, but now *we are running out of food*’. (Male, age 17)

The second most frequently endorsed category was stress, reported by 36% of participants of whom 65% reported feeling stress in the moderate to high range (see Table 2). Four categories were identified in this domain including: (1) distress about the future, endorsed by 30% of participants reporting stress; (2) frustration, endorsed by 28% of participants reporting stress; (3) boredom, endorsed by 26% participants reporting stress; and (4) increased work/homework, endorsed by 15% of participants reporting stress. Participants specifically reported feeling stressed as a result of being bored during the the lockdown period, frustration with lockdown restrictions as well as taking frustration out on others, and struggling with increased amounts of homework, work or overseeing the increased homework of their children (see Table 3). Participants explained: ‘School work seems endless and excessive and takes much longer to get done’ (Female, age 29); and

‘I have felt very bad because lately *my parents have been reprimanding me for everything.* Sometimes I believe I am the one doing the wrong things, but I realize that *they get angry even over the smallest things*. I am trying to be understanding because we are going through a difficult situation in my house. Due to the pandemic, my parents were forced to sell some of our belongings because *we do not have enough money to survive*. I want to help out economically, but I have not been able to get a job yet. At times *I feel really frustrated because I cannot help them as I would* . . . I try to be understanding when they get frustrated and angry. However, *it hurts that I can’t please them and that they scold me* all the time’. (Female, age 21)

Participants also reported distress over the future and not knowing what is to come (i.e. when the pandemic will end, if it will get worse before it gets better).

The third most frequently endorsed category was exacerbation of pre-Covid-19 mental health issues, expressed by 19% of respondents of whom 48% rated these symptoms as moderate to severe (See Table 2). Relational problems accounted for the majority of pre-exiting issues, endorsed by 63% of participants reporting an increase in pre-existing symptoms/mental health issues. These included separations from partners; poor family communication; conflict/fighting with family; stressors related to family members (i.e. estrangement, teen pregnancy, children with behavioral problems); and interrupted grief. Intrapersonal factors accounted for the remaining issues, endorsed by 23% participants reporting increased pre-existing symptoms/mental health issues. Issues noted in this area included, low self-esteem, anxiety, irritability (see Table 3). Several participants noted that a loved one had died prior to the pandemic so when the pandemic began they were already feeling sad and not only was their grieving was interrupted but the person they would normally turn to in times of stress was no longer there to support them, which makes them feel even worse. Others commented that being in the lockdown period with family member they didn’t get along with well to begin with has led to even more arguments and disagreements in the house resulting in increased stress, irritability, and tension.

Depression and feelings of burnout were equally reported by 12% of participants, with 18% of those participants rating the severity of these symptoms as moderate to high (See Table 2). Qualitative analysis revealed three category related to feelings of depression, specifically (1) sadness; (2) loss of routines; and (3) insomnia. One core category related to burn-out was also identified, specifically, lack of motivation to complete tasks (see Table 3). Comments from participants reporting depression included:

‘I know that *everything that is happening to me is not normal*. I am a primary school teacher; I used to work from 6 am until 6 pm. The school where I work is a couple of hours away in another municipality. *My routine has completely changed.* I am now at home trying to do the cleaning, but lately I have not been well and it is due to the pandemic. *There are days that I just want to run away* when I start to think about the situation. *Sometimes I cannot sleep*. I feel that everything is wrong for me and everything irritates me, so *I try to be alone*. I have 2 daughters and sometimes they want to play, but *I am not in a good mood and don’t want to play with them*. My wife does not understand me and I try not to tell her anything. *I do not deny that I have had a different attitude with my family and it is because of everything I feel*. Sometimes *I feel like crying* because I don’t know what to do, but I try each day to be better. I feel that I need to let off steam for everything I feel and maybe that way I will feel better because my wife does not understand what I feel’. (Male, age 42)

‘I don’t like talking about these things very much, it is not easy to say these things out loud and trust people, but with all that has happened to me *I have felt discouraged*. It has been difficult for me and my sister. I don’t know how it impacts my mom because she avoids talking about it, but maybe it is difficult for her too. Sometimes I feel that with all of it, I am not the same’. (Male, age 18).

Participants experiencing burnout commented, ‘Everything seems to take longer to do and I’m tired of all the housework’ (Female, 25), and ‘It seems there is some more to do know and I’m overworked’. (Male, age 29)

A very low number of participants identified a decrease in their sense of personal safety during the pandemic (2%); or specific incidents of domestic violence in their homes (3%). Participant stated concerns regarding increased violence by family members in their household. One participant shared worries about her neighbors and suspected domestic violence in the neighbor’s family (Table 4).

**Table 4.** Sense of safety.

Safety/security	Participants identifying related issues *n* (%)
Increased fears about safety at home	5 (2)
Increased incidence of domestic violence	9 (3)

## Discussion

This is the first study to examine the mental health impact of the Covid-19 pandemic on highly vulnerable communities across Guatemala. We aimed to identify the psychosocial factors contributing to psychological distress experienced during the lockdown period related to the pandemic among individuals residing in marginalized, high-risk communities. Our findings indicate that a concerning number of people have experienced moderate to high levels of anxiety and stress and an increase in pre-exiting mental health symptoms since the onset of the pandemic.

We found that almost half of the participants (46%) endorse experiencing anxiety and over two-thirds endorse experiencing stress since the onset of the pandemic. Although no studies have specifically examined the association between the pandemic and rates of anxiety in low- and middle-income Latin American countries, this finding is consistent with recent studies from other areas of the globe (Antunes et al., 2020; Barzilay et al., 2020; Hyland et al., 2020; Kuang et al., 2020; Malesza & Kaczmarek, 2021; Verma & Mishra, 2020; Wong et al., 2020). Traumatic events, such as the pandemic, can have adverse effects on individuals’ mental health and decrease their feelings of safety and security. Uncertainty in multiple areas of life including, when the pandemic will end; when a treatment or vaccine will become available; whether rates of the virus will increase before they decrease; constant exposure to news regarding death and infection rates; increased social isolation due to shelter-in-place orders, quarantines, and lockdown restrictions, has been shown to result in increased anxiety, worry, and stress (Brooks et al., 2020; Neria & Sullivan, 2011; Özdin & Bayrak Özdin, 2020; Salari et al., 2020; Torales et al., 2020; Wang et al., 2011; Wang, Zhang, et al., 2020).

Evidence-based practices for treating anxiety and stress often involve focusing on irrational beliefs related to fear, danger, safety, and security and challenging cognitive distortions such as catastrophizing, over-generalizing, and jumping to conclusions (Kaczkurkin & Foa, 2015; Otte, 2011). However, given the uncertainty regarding Covid-19, particularly around means of transmission, rate of spread, and when it will resolve, and the overall novelty of the illness leading to a general lack of understanding regarding the nature of the disease, it is difficult to gather the evidence needed to counter these beliefs and develop rational, balanced alternatives. This suggests that new strategies for addressing pandemic related anxiety and stress may be needed to effectively address the increasing challenges individuals are experiencing.

We also found an increase in pre-existing mental health symptoms since the onset of the pandemic. It has been noted that even under normal circumstances, individuals with mental illness have a lower life expectancy and poorer physical health than the general population (Rodgers et al., 2018). Therefore, it is likely that individuals with pre-existing mental health conditions will not only be at increased risk of Covid-19 infection, but will also experience increased risk of negative psychological effects related to the pandemic (Cullen et al., 2020; Taylor, 2019). Indeed, increased severity of pre-existing mental illness has been associated with the current pandemic in other studies (Chatterjee et al., 2020; Duan & Zhu, 2020). Outreach strategies for identifying individuals struggling with pre-existing mental health issues will be critical for ensuring those at most risk get the treatment needed to address their mental wellbeing. This should include enhanced public health efforts to increase awareness and encourage help-seeking and improved access to psychological services. Although remote/virtual technologies for mental health service delivery have proliferated during the pandemic, access barriers to these mobile technologies for those living in highly vulnerable communities is still an issue. In the Red Zone districts of Guatemala, for example, where economic and resources and mental health services are scarce, internet service and stable Wi-Fi is limited, in general, and access to computers in a private setting that would facilitate a virtual treatment session, a proper use of technology to enhance public health knowledge and expand services might be particularly challenging. Mobile phones, however, are widely available. Text-based services and mobile applications may prove a more effective avenue for such outreach and intervention efforts.

We found that only a small percentage of participants endorsed feeling depressed as compared to anxious or stressed, despite sharing symptoms associated with depression such as sadness, irritability, difficulty sleeping, lack of self-esteem, and despite experiencing stressors that are often associated with depression such as loss and separation. This may be partially attributable to the increased stigma associated with depression compared to anxiety and stress and participants being less willing, therefore, to disclose these feelings. Research has found that individuals with higher levels of perceived public stigma are further influenced by negative stereotypes related to treatment and are less likely to see the importance of seeking professional psychological help. Therefore, if a person is fearful of being stigmatized, he or she may be reluctant to seek out and attend mental health services (Corrigan & Matthews, 2003; Pattyn et al., 2014).

Stigma has been defined as being comprised of two key dimensions: public stigma and self-stigma. Public stigma refers to negative attitudes (prejudice), beliefs (stereotypes), and behavior (discrimination) toward stigmatized individuals. Self-stigma refers to the internalization of these experiences by the stigmatized individual (Corrigan & Watson, 2002). Prior research has found that public stigma regarding depression is stronger than it is toward anxiety (Wood et al., 2014). When compared to depression, for example, the public views anxiety more favorably, holds less negative stereotypes about it, and is considers anxiety much more likely to overcome. Conversely, more negative stereotypes are held regarding depression and individuals who experience depression are often labeled as lazy and difficult engage with (Thornicroft et al., 2007). One study that examined correlates of stigma associated with depression found that severity of depression symptoms predicted higher self-stigma, indicating that those with more severe depression hold more stigmatized views of themselves (Yen et al., 2005). In comparison, other research focused on anxiety has found that greater levels of anxiety symptoms did not predict higher levels of either perceived or personal stigma toward anxiety (Busby Grant et al., 2016).

Although limited and largely based on Latinos in the US, evidence does suggest that mental health stigma is a negative predictor of help-seeking among Latinos (Abdullah & Brown, 2011; Brennan et al., 2005). Latino cultural values also may serve as a disincentive toward formal mental health treatment given the cultural belief that psychological issues should be resolved by oneself or within one’s family.

As such, a Latino individual experiencing symptoms of psychological distress may feel that seeking treatment for their problems could bring shame or embarrassment to the family (Cheng et al., 2013; Rastogi et al., 2012), or that he or she is unworthy of dignity and respect (Abdullah & Brown, 2011). For example, a study of Latino families in which a family members has a severe mental illnesses found that stigma was the most commonly reported barrier to seeking treatment, with formal treatment being associated with shame about one’s mental health and how it reflects upon the family (Marquez & Ramírez García, 2013).

Other factors contributing to self-identifying depression may relate to our findings of very low self-reports of family violence. Individuals experiencing family violence experience higher rates of depression often linked to the normalization of violence and the self-attribution of guilt. (Karakurt et al., 2014). As noted by a Guatemalan court judge (Mercier, 2020), family violence is normalized, preventing people directly affected from identifying it as a crime (and reporting it). This normalization is directly supported by the outrageous rates of impunity for any domestic violence cases reaching the justice system.

Despite existing data on the alarming rates of violence against women, domestic violence, and community violence in Guatemala (Mercier, 2020; UNHCR, 2015; WHO, 2013), and several recent studies identifying the increased risk of domestic violence during lockdown measures both globally (Sacco et al., 2020; Sharma & Borah, 2020), and in Guatemala (Gamez, 2020; Stephen, 2020), fewer than 5% of participants endorsed concerns about safety or increased violence. And, this in the context of health care professionals and social workers identifying domestic violence and community violence as major concerns for the communities involved in this study. Along with concerns about child abuse that cannot be monitored and/or detected during the pandemic as children are confined to their homes (UN News, 2020; Usher et al., 2020), further exploration of issues related to safety and violence across vulnerable communities is imperative.

Lastly, the small but notable endorsement of feelings of burnout is important to acknowledge. Most research examining burnout during the pandemic has focused on frontline works, primarily health, and mental health care workers (Greenberg et al., 2020; Heath et al., 2020; Sriharan et al., 2020). Far fewer studies have examined burnout in the general population as a result of lockdowns and shelter-in-place orders. The little research examining parental burnout suggests that the significant disruption in the daily routines of families related to work, school, and extracurricular activities, and stressors related to economic, educational, and health concerns all in the context of limited social support has taken a toll on the mental health of parents resulting in parental burnout (Griffith, 2020). Research has found that parents who report burnout report a negative impact on their relationship with their partner and on the health and wellbeing of their children (Heath et al., 2020; Mikolajczak et al., 2019). Our study extended these findings and suggests that even those who are not parents report increased burnout related to work in and outside of the home, including an inability to concentrate and complete tasks, lack of desire to complete required work activities, and inability to keep up with needed tasks, and a lack of motivation resulting in feelings of being overwhelmed, overworked significantly impacting feelings of self-efficacy.

### Strengths and limitations

This study has a number of strengths. To begin, this is the first study to give voice to this often overlooked, under-represented population. Also, we were able to achieve a high rate of participation, largely attributable to the positive, long-standing presence of HRI in the community, and its well-established relationships with other NGO’s and organizations on the ground in the communities surveyed. Further, all of the callers utilized a semi-structured interview schedule and received training on semi-structured interviewing techniques to ensure consistency among interviewers and interviews, while still allowing for flexibility in data collection. Additionally, there was no fixed length assigned to the interviews, which allowed caller to first develop rapport with participants and to allow for participants to elaborate on the experiences that were important to them. The downside of such flexibility is that there was some level of variation in the depth of detail across interviewers’ reports and notes accompanying each call.

There are some other methodological limitations worth mentioning. We focused on communities in which economic, health, and mental health resources are extremely limited and rates of poverty, crime, and violence are high. These findings may not present a national perspective or be generalizable to communities that are not as highly vulnerable. Further, our sample was approximately two-thirds female, which may have had an impact on the findings given cultural norms regarding gender roles and expectations. Additionally, we used cross-sectional data and employed qualitative analyses. While this allowed us to better understand the perspective of the individual experiencing psychological distress, quantitative studies are needed to further identify risk and protective factors and prospective studies are needed to understand the impact of the pandemic over time and changes in mental health status over the course of prolonged lockdowns and after restrictions are lifted. Also, a follow-up study including more in-depth interviews with selected participants might provide additional information, particularly on more sensitive topics, such as depression rates and intensity, and domestic violence and community safety. Additionally, data collection took place over the course of several months during which infection and death rates may have varied. This could have had an impact of respondents’ mental health. Future prospective studies should address changes in mental health status over time. Finally, as is the case with all qualitative studies, there is a degree of interpretation inherent in our findings. However, established guidelines for ensuring rigorous methodology in the qualitative analysis was followed.

## Conclusion

Due to the unanticipated nature of the pandemic and the ensuing taxing measures such as lockdowns, quarantines, and stay-at-home orders, the Covid-19 pandemic has taken a significant toll on mental well-being across already high-risk populations such as those residing in highly vulnerable communities across Guatemala. The findings of our study highlight the urgent need for community capacity-building to develop and sustain prevention and intervention services to address mental health needs of individuals in these communities that are sorely lacking in resources to address the growing mental health needs resulting from the pandemic. Further studies focusing on individuals’ and communities’ sense of safety, and the impact of the pandemic on domestic violence and community violence are essential for creating safe spaces that will allow better and more timely responses to health and mental health care needs of these communities. Lastly, efforts to engage individuals in mental health services should include a focus reducing stigma and raising public awareness around mental health and depression, in particular.
